# Withdrawal: Sirtuin 1-mediated deacetylation of XPA DNA repair protein enhances its interaction with ATR protein and promotes cAMP-induced DNA repair of UV damage

**DOI:** 10.1016/j.jbc.2020.100185

**Published:** 2021-03-27

**Authors:** Stuart G. Jarrett, Katharine M. Carter, Robert-Marlo Bautista, Daheng He, Chi Wang, John A. D'Orazio

VOLUME 293 (2018) PAGES 19025–19037

This article has been withdrawn by the authors except Stuart Jarrett, who could not be reached. The withdrawing authors were unable to provide original data corresponding to many of the micrographs presented in the article according to Journal policy. In Fig. 3*D*, the PLA images for XPA-WT, XPA-K63Q, and XPA-K67Q were duplicated. Also in Fig. 3*D*, the DAPI merged images for XPA-WT and XPA-K63Q were duplicated.

